# Effects of Dietary Medlar (*Mespilus germanica* L.) Extract on Growth Performance, Innate Immune Characteristics, Antioxidant Status, and Responses to Crowding Stress in Rainbow Trout (*Oncorhynchus mykiss*)

**DOI:** 10.1155/2023/7613330

**Published:** 2023-08-02

**Authors:** Indrajit Patra, Afiska Prima Dewi, Mohammed Fawzi, Fadhil Hussam, Israa K. Obayes, Mohammed Ahmed Jamal, Hayder A. Hammoodi, Zainab R. Abbass, Mahnaz Dadras, Fariborz Narimanizad

**Affiliations:** ^1^NIT Durgapur, Durgapur, West Bengal, India; ^2^Department of Nutrition, Faculty of Health, Universitas Aisyah Pringsewu, Lampung, Indonesia; ^3^Al-Manara College for Medical Sciences, Amarah, Iraq; ^4^College of Medical Technology, Medical Lab Techniques, Al-Farahidi University, Baghdad, Iraq; ^5^Medical Laboratory Techniques Department, AL-Mustaqbal University College, Hilla, Iraq; ^6^Al-Nisour University College, Baghdad, Iraq; ^7^College of Pharmacy, Al-Ayen University, Thi-Qar, Iraq; ^8^Department of Pharmacy, Al-Zahrawi University College, Karbala, Iraq; ^9^Department of Fisheries, Faculty of Natural Resources, University of Tehran, Karaj, Iran

## Abstract

High stocking density is a stress factor that potentially affects physiological and immune responses. In this study, the effects of medlar (*Mespilus germanica*) extract (ME) supplementation on growth performance, antioxidant, immune status, and stress responses in rainbow trout (*Oncorhynchus mykiss*) were studied. Six hundred fish (40.19 ± 1.09 g; average fish weight ± standard error) were distributed randomly into five experimental groups (assayed in triplicates). The experimental diets were formulated as follows: 0 (T1, control), 0.5% (T2), 1% (T3), 1.5% (T4), and 2% (T4). After 60 days feeding trial, the fish were confined, and the density increased (60 kg/m^3^) for further 14 days. Results showed significant increases in final weight (FW), weight gain (WG), specific growth rate, and feed intake in the T4 compared to the control (*P* < 0.05). The feed conversion ratio (FCR) in T4 significantly decreased compared to the control (*P* < 0.05). Also, the treated groups showed significant improvements in catalase (CAT), superoxide dismutase (SOD), glutathione peroxidase (GPx), lysozyme (LYZ), total immunoglobulin (total Ig), respiratory burst activity (RBA), total protein, and phagocytosis (PHA) (*P* < 0.05). Moreover, compared with the control group, supplementation could significantly decrease glucose (GLU) and cortisol (CORT), alanine transaminase (ALT), lactate dehydrogenase (LDH), aspartate transaminase (AST), and alkaline phosphatase (ALP) (*P* < 0.05). After the challenge, FW and WG in all treated challenge groups were significantly improved compared to the control group (*P* < 0.05). FCR showed a significant decrease in all treated challenged groups compared to the control group (*P* < 0.05). However, malondialdehyde, CAT, GPx, SOD, LYZ, complement activity (C3 and C4), total Ig, RBA, peroxidase, and PHA in challenged treated groups were significantly increased compared to the control group (*P* < 0.05). All treated challenged groups showed lower ALT, LDH, AST, ALP, GLU, and CORT levels than the control group (*P* < 0.05). The experiment herein successfully demonstrated that dietary ME stimulated fish growth, antioxidant status, and immune responses in crowding conditions and can be recommended as beneficial feed additives for rainbow trout.

## 1. Introduction

Stressors generally challenge the various physiological functions due to recovering the homeostatic status of fish. Besides, elevating the innate immune mechanism, health condition, and growth rate of the host is an ideal approach to disease and stress management strategies in modern aquaculture [1, 2].

Nutritional treatment has been recommended as a useful method to mitigate fish stress [3]. Immunostimulants are considered imperative alternatives and unconventional compounds that modulate the immune system of the hosts to increase resistance against free radicals and pathogens [4, 5]. Medicinal plant extracts, rich in bioactive ingredients, are known as eco-friendly natural and functional immunostimulants. Herbal supplements represent an alternative to traditional antimicrobials, which influence immunocompetency in different fish species of interest in aquaculture [6, 7].

One of these medical herbs is medlar (*Mespilus germanica* L.) belongs to the Rosaceae family and mainly grows in the temperate regions of southwest Asia and southeastern Europe [8]. Medlar fruit gained value in human consumption and commercial importance in recent years [9]. The fruit is often consumed or sold in the local markets and stores. Medlar fruit is widely consumed in some countries such as Turkey, a unique place where the people grow the wild and alternative cultivars for the consumption of the fruits [10]. Fruit, leaf, stem bark, seed, and wood of medlar can be used as herbal medicine [8]. Medlar fruit and leaves can inhibit reactive oxygen species (ROS) due to the presence of flavonoids, polyphenol oxidase, antibiotics (genipic acid and genipinic acid), and minerals. Therefore, medlar contains antibacterial and antioxidant properties [10, 11].

One of the freshwater aquaculture species commercially farmed under intensive or semi-intensive systems is rainbow trout in Iran, *Oncorhynchus mykiss* [12]. High stocking density causes stress and widespread infectious diseases resulting in significant economic losses [13]. The present study was conducted to examine the effect of dietary medlar (*M. germanica* L.) extract (ME) on growth performance, antioxidant, and immune responses of rainbow trout exposed to crowding stress. To the best of our knowledge, little information is available about the effect of medlar extract on rainbow trout immunity.

## 2. Material and Methods

### 2.1. Herbs' Extract

Fresh leaves of medlar were purchased from a local market in Rasht, Iran. Leaves were washed using sterile distilled water and oven-dried at 50°C for 48 hr. 80% ethanol was added to 200 g dried medlar and then incubated in a shaker incubator for 24 hr. The extract was filtered through a Whatman paper (no. 1) [14]. The alcohol was removed using a rotary evaporator at 40°C, concentrated extract, and stored at 4°C until use [15, 16].

#### 2.1.1. Total Phenolic

Total phenol content was determined based on the Folin–Ciocalteu method. Briefly, a 120 *μ*l aliquot of the dissolved extract was mixed with 50 *μ*l Folin–Ciocalteu 10% reagent. Then, 30 *µ*l Na_2_CO_3_ 20% was added and incubated for 1 hr at 37°C; the absorbance was measured at 735 nm in the dark and compared to the calibration curve of gallic acid [17]. The results were expressed as mg of gallic acid equivalent (GAE)/g.

#### 2.1.2. Flavonoids Content

Total flavonoid content was measured using aluminum chloride [18]. In this method, 250 *μ*l extract was added to 1,250 *μ*l of distilled water, and then 75 *μ*l of 5% sodium nitrate solution was added. After 5 min, 150 *μ*l of 10% aluminum chloride solution was added and kept at room temperature for 5 min. Finally, 500 *μ*l of sodium hydroxide solution and 775 *μ*l of distilled water were added to the mixture. The mixture was homogenized, and the adsorption rate was read at 510 nm. A standard quercetin curve was used, and the results were expressed in mg of quercetin equivalent (QE)/g.

#### 2.1.3. DPPH Radical Scavenging Capacity

About 0.2 ml of 0.1 mM 1, 1-diphenyl-2-picrylhydrazyl (DPPH) ethanol (150 *μ*M) was added to the 100 *µ*l of seaweed extract. The sample was then incubated for 30 min at room temperature. The absorbance was measured at 520 nm. The radical scavenging activity of DPPH was calculated using the following formula [19]:

(1) $$\begin{array}{c} \%\text{inhibition}=100\left(A_{blank}-A_{sample}\right)/A_{blank}. \end{array}$$

#### 2.1.4. *β*-Carotene/linoleic Acid Assay

The inhibition of linoleic acid peroxidation was determined according to Miller [20]. About 20 mg *β*-carotene was dissolved in 1 ml of chloroform. *β*-Carotene methanolic solution (28 *μ*l) was homogenized with linoleic acid (28 *μ*l) and Tween 40 (200 mg). After evaporation of chloroform using rotary at 40°C, 50 ml of distilled water was added. The same procedure was repeated using butylate hydroxy anisole as the antioxidant standard. Readings were performed every 15 min at 470 nm. Where Ac and As are the absorbances of the control and samples, respectively. The potency of essential oil oxidation was calculated based on the following formula:

(2) $$\begin{array}{c} \begin{array}{c} \text{Ac}=\text{Initial absorbance}-\text{final absorbance},\\ \text{As}=\text{Initial absorbance}-\text{final absorbance}\\ \%I=\left(Ac-As/Ac\right)\times 100. \end{array} \end{array}$$

### 2.2. Preparation of Experimental Diet

There were four diets in this experiment, containing 0 (T1, control), 0.5% (T2), 1% (T3), 1.5% (T4), and 2% (T5) ME. Such concentrations were chosen based on a previous study [21]. The ingredient and proximate composition of basal diet are shown in Table 1. Enough distilled water was added to the feed ingredients and thoroughly mixed. The dough was then passed through a meat grinder to obtain equal size particles. Finally, the pellets were dried at room temperature. Dried pellets were stored in plastic bags at 4°C until use. Experimental diets were analyzed for proximate chemical composition [23].

### 2.3. Fish and Maintenance Conditions

Rainbow trout were cultured in spring water on a local farm, and the water quality parameters, including temperature, dissolved oxygen, pH, and unionized ammonia nitrogen, were monitored daily at 14–15°C, and 7.5–8.2, 7.1–7.3, and 0.002 mg/l, respectively [22]. Water temperature, dissolved oxygen, and pH were determined by portable apparatus (Hach HQ40d, Loveland, Colorado, USA). Also, ammonia was determined by a digital photometer (Wagtech 7,100, Berkshire, UK). After acclimatization, 600 apparently healthy rainbow trout juveniles (average weight of 40.19 ± 1.09 g; mean ± SE (standard error)) were allocated to 15 fiberglass tanks containing 300 l of aerated water at a density of 40 fish/tank (30 kg/m^3^). The experiment lasted for 60 days and five test groups in triplicates. Fish were fed based on *ad libitum*, three times daily [24]. After the 60 days of rearing, the fish final weight (FW), survival rate (SR), and feed conversion ratio (FCR) were determined, and the fish density increased to 60 kg/m^3^ for further 14 days [25]. After the stress, SR and growth parameters were determined in all treatments. Besides, blood samples were taken from all treatments before and after the crowding stress. In the stress period, temperature, dissolved oxygen, pH, and unionized ammonia nitrogen levels were 14.3–15.2°C, 6.9–7.4, 7.2–7.4, and 0.005 mg/l, respectively [25, 26].

### 2.4. Growth Performance

Growth performance and survival parameters were calculated using the following equations [22]:

(3) $$\begin{array}{c} \text{Weight gain}\left(\mathrm{WG};g\right)=\text{final bodyweight}-\text{initial bodyweight}, \end{array}$$

(4) $$\begin{array}{c} \text{Specific growth rate }\left.\left(\mathrm{SGR};\%\text{/}{\text{day}}^{-1}\right)=\left(\left(\ln\left(\text{final bodyweight}\right)-\ln\left(\text{initial bodyweight}\right.\right)\right)/\text{trial period}\right)\times 100, \end{array}$$

(5) $$\begin{array}{c} \text{Feed intake}\left(\mathrm{FI},g/\text{day}\right)=\text{total consumed feed per fish}/\mathrm{day}, \end{array}$$

(6) $$\begin{array}{c} \text{Feed conversion ratio}\left(\mathrm{FCR}\right)=\text{feed intake}\left(g\right)/\text{weight gain}\left(g\right), \end{array}$$

(7) $$\begin{array}{c} \text{Survival rate}\left(\text{SR};\%\right)=\left(\text{final number of fish}/\text{initial number of fish}\right)\times 100. \end{array}$$

### 2.5. Sampling

After the experimental time, the fish were fasted for 24 hr and anesthetized (75 mg/l eugenol) for 1 min [25]. Blood was withdrawn from the caudal vein and was divided into two parts; heparin-coated tubes used for respiratory burst activity (RBA) and phagocytosis (PHA) and nonheparin tube for serum parameters. Serum samples were separated at 300 g for 15-min centrifugation. The supernatant was collected in a fresh sterile tube and stored at −80°C [27].

### 2.6. Antioxidant Status

Malondialdehyde (MDA, *μ*M/l) content was determined with the thiobarbituric acid reaction by a commercial kit (ZellBio GmbH, Veltinerweg, Germany). Superoxide dismutase (SOD) was defined as the quantity of enzyme (U/ml) that inhibits the reduction of oxidase-cytochrome C (ZellBio GmbH, Veltinerweg, Germany). Catalase (CAT, U/ml) was utilized to decrease H_2_O_2_ absorbance according to Góth's [28] method. Serum glutathione peroxidase (GPx) activity was measured based on the conversion of glutathione to glutathione disulfide using a commercial kit (Zellbio®, Berlin, Germany), as suggested by Hoseini et al. [29].

### 2.7. Enzymatic Assay

Serum enzymes lactate dehydrogenase (LDH), alkaline phosphatase (ALP), alanine aminotransferase (ALT), and aspartate aminotransferase (AST) activity were detected by biochemistry analyzer, using commercial kits (Pars Azmun Co., Tehran, Iran) [30].

### 2.8. Immunological Analysis

Lysozyme (LYZ, U/ml) activity was measured through turbidometric assay and lysis of *Micrococcus luteus* in 0.2 mg/ml in a 0.05 M sodium phosphate buffer (pH = 6.2) as the substrate according to the method of Ellis [31]. The complement activity (C3 and C4 (mg/dl)) was measured using ELISA (ELX800, BioTek, Vermont, USA) and a commercially available kit (Pars Azmun Co., Tehran, Iran). Total immunoglobulin (total Ig, mg/dl) activity was measured based on the total protein (TP) concentration of the samples before and after precipitation in polyethylene glycol. The RBA (RLU/S) was measured by chemiluminescent assay (LUMI Skan Ascent T392, Finland) as previously described by Binaii et al. [32].

The peroxidase (PRO, *µ*g/ml) activity in serum or leukocytes was measured according to Cordero et al. [33]. Briefly, 5 *µ*l of serum was diluted with 45 *µ*l of Hank's Balanced Salt (without Ca^2+^ or Mg^2+^) in flat-bottomed 96-well plates. About 100 ml of the solution, including 40 ml distilled water, one pill of 3, 30, 5, 50- tetramethylbenzidine (TMB, Sigma), and 10 *µ*l H_2_O_2_ were added. The color-change reaction happened in the 30–60 s. Then, 50 *µ*l of 2 M sulfuric acid (2 M H_2_SO_4_) was added. Finally, the optical density was read at 450 nm in a plate reader. Standard samples without serum were used as blanks.

Leukocyte PHA (%) activity was determined with yeast as an indicator according to the Zhou et al. [34] method. Dried live yeast (*Saccharomyces cerevisiae*, Baker's yeast) was incubated in 2% sucrose (pH = 3–4) at 30°C for 2 hr, then boiled for 30 min. The yeast was centrifuged (800 g for 10 min) and washed twice with 0.85% NaCl (concentration of 2 × 10^8^ cells/ml). Approximately 40 *µ*l of blood heparin and 20 *µ*l suspension were collected in the microtube and were incubated in a shaker incubator at 30°C for 30 min. Finally, dried smears were stained with Wright–Giemsa. One hundred phagocytoses were analyzed per slide under the optical microscope by using the below formula:

(8) $$\begin{array}{c} \text{Percentage phagocytosis}\left(\%\right)=100\%\times \left(\text{number of phagocytic cells with swallowed yeast cells}/\text{number of phagocytes enumerated}\right). \end{array}$$

### 2.9. Biochemical Analysis

Cortisol (CORT, ng/ml) concentration in serum samples was quantified using an ELISA kit (IBL Co., Gesellschaft für Immunchemieund Immunbiologie, Germany). Serum glucose (GLU, mg/dl) content was measured by a commercial kit (Pars Azmun Co., Tehran, Iran) [30].

### 2.10. Statistical Analysis

Before performing the analysis of variance (ANOVA), the normality of the data was checked using the Kolmogorov–Smirnov test, and Levene's test was used to assay the variances of data. Statistical analysis of data performed using SPSS software version no. 20.00 (SPSS Inc., Chicago, IL, USA), and the results represent the mean ± SE. Differences in studied parameters among the experimental groups were processed by one-way ANOVA followed by Tukey's multiple comparison test considering *P* < 0.05 as the significance level.

## 3. Results

### 3.1. Herb

Bioactive compounds such as total polyphenol content (189.22 ± 6.22 g mg GAE/g), total flavonoids (57.55 ± 5.31 mg QE/g), and antioxidant capacities evaluated by DPPH (56.77 ± 3.66%), and *β*-carotene/linoleic acid (74.11 ± 4.91%) of ME are shown in Table 2.

### 3.2. Growth Performance

Table 3 represents the growth performance parameters of rainbow trout fed the control and supplemented diets. After 60 days of the feeding trial, results revealed that administration of 1.5% ME (T4) significantly increased FW, WG, and SGR and decreased FCR value compared to the control (*P* < 0.05). However, no statistical variations were recorded in FI and SR in fish-fed supplemented diets and the control group (*P* > 0.05).

After the challenge, 1%, 1.5%, and 2% (T3, T4, and T5) ME significantly improved FW compared to the control group (*P* < 0.05). Determination of WG showed a significant increase in all experimental treatments over the control treatment (*P* < 0.05), whereas FCR analysis showed a significant decrease in experimental treatments over the control treatment (*P* < 0.05). 1.5% and 2% ME significantly increased FI. No significant differences were observed for the SGR and SR between the fish-fed dietary ME and the control group (*P* > 0.05).

### 3.3. Antioxidant Status

Table 4 represents measured values of antioxidant status. Based on our results, no significant difference was observed in the MDA level between the treatments (*P* > 0.05). The value of SOD and CAT increased in T3 and T4 compared to the control group (*P* < 0.05). Other treatments displayed no significant difference compared to the control (*P* > 0.05). In addition, GPx analyses indicated a significant increase in all ME treatments compared to the control group (*P* < 0.05).

After the challenge, the MDA value didn't influence in T2 and T3 groups (*P* > 0.05); however, significant decreases were observed in other treatments compared to the control group (*P* < 0.05). Also, the SOD, CAT, and GPx activities in fish fed T3, T4, and T5 were significantly increased than that in the control group (*P* < 0.05).

### 3.4. Enzyme Parameters

Enzyme parameters of rainbow trout fed different levels of ME are presented in Table 5. Compared with the control group, T3, T4, and T5 had significantly lower ALT activity (*P* < 0.05), however, the T2 displayed no significant difference (*P* > 0.05). AST contents in fish fed 1.5% (T4) ME were significantly lower than those in fish fed the control diet (*P* < 0.05). Notably, there were no significant differences between the other groups (*P* > 0.05). Moreover, the value of ALP decreased in fish groups fed the supplemented diets compared to the control group (*P* < 0.05). ME administration significantly decreased LDH levels in T3, T4, and T5 compared to the control group; the lowest level of LDH was recorded in T3 (*P* < 0.05).

After the challenge, the value of ALT decreased in fish groups fed the supplemented diets, compared to the control group (*P* < 0.05). Moreover, all experimental groups showed lower AST levels than the control group (*P* < 0.05), though no statistical variations were recorded among them (*P* > 0.05). Compared to the control group, ALP and LDH contents were significantly lower in all treated groups. The lowest level of them was found in the T4 (*P* < 0.05).

### 3.5. Immune Analysis

The effect of dietary ME on the immune parameters of rainbow trout is presented in Table 6. LYZ levels were significantly higher in the T3 and T4 compared to the control group (*P* < 0.05); other treatments (T2 and T5) had shown no significant difference compared to the control treatment (*P* > 0.05). No statistical variations were observed in C3 and C4 values between the fish-fed experimental groups and the control treatment (*P* > 0.05). Significantly higher total Ig level was found in all experimental treatments compared with the control groups (*P* < 0.05); however, there were no significant differences between them (*P* > 0.05). Moreover, results exhibited a significantly higher amount of RBA in fish-fed ME diets than in the control group (*P* < 0.05). Fish treated with T3, T4, and T5 have significantly higher PRO activity than T1 (*P* < 0.05). Besides, T2 had shown no significant differences than T1 (*P* > 0.05). The highest level of PHA was recorded in the T4 group (*P* < 0.05); other treatments showed no significant difference than that of the control (*P* > 0.05).

After the challenge, results exhibited the most significant amount of LYZ and C4 in the fish-fed T4 diet than in the control group (*P* < 0.05). The other treatments displayed no significant differences. Additionally, the serum total Ig, RBA, PRO, and PHA levels were significantly higher in the T3, T4, and T5 compared to the control group (*P* < 0.05), T2 had shown no significant difference compared to the control treatment (*P* > 0.05).

### 3.6. Biochemical Analysis

**Figure 1:**
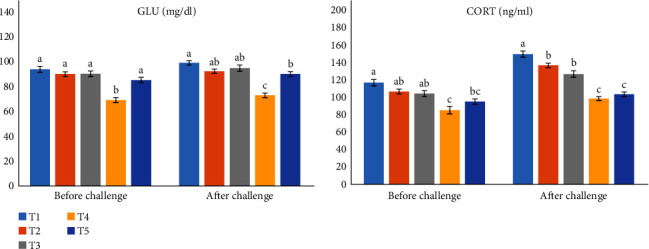
Serum glucose and cortisol levels of rainbow trout (*Oncorhynchus mykiss*) fed different levels of dietary medlar (*Mespilus germanica* L.) extract under crowding stress. Values are presented as the mean ± SE (*n* = 3). Different letters (a–c) in each row indicate statistically significant differences (*P* < 0.05). T1 (0, control); T2 (1%); T3 (2%); T4 (3%); T5 (4%) ME. GLU, glucose; CORT, cortisol.

According to Figure 1, GLU levels were significantly lower in the T4 compared to the control group (*P* < 0.05); other treatments had shown no significant difference. CORT assessment showed a significant decrease in T4 and T5 compared to the T1 (*P* < 0.05); meanwhile, no significant differences were observed among other dietary groups (*P* > 0.05).

After the challenge, the GLU both in T4 and T5 were recorded as significantly lower than the control group (*P* < 0.05). Moreover, the value of CORT decreased in fish groups fed different doses of ME diets compared to the control group (*P* < 0.05).

## 4. Discussion

Stressors such as overcrowding depend on the duration and intensity and can cause acute or chronic stress responses [35]. Developing medicinal plants as alternative strategies incorporated in diet formulations has become a major trend in the last decade to improve the immune system and increase resistance to environmental stresses [2, 36] The present study indicates that supplementation of ME in rainbow trout diet improved the growth performance, antioxidant, and immunological parameters, as well as modulate the immunity in response to crowding stress. To the best of our knowledge, this is the first investigation that looks at the effect of ME on rainbow trout immunity and stress responses.

The findings of this study showed that before the challenge, supplementing the diet with 1.5% ME had growth-promoting effects (FW, WG, SGR, and FCR) on rainbow trout, whereas at high stocking density, 1%, 1.5%, and 2% ME increased fish growth performance compared to the control group. Although the exact mechanism for this effect is not clear, ME may have beneficial effects to reduce stress detrimental effects. Daily FI and growth rate reduction at short-term crowding stress were observed on several species, such as European seabass (*Dicentrarchus labrax*) [37] and gilthead seabream (*Sparus aurata*) [38]. High stocking density, as a chronic stressor, reduces food consumption due to poor water quality, social behavior alterations, and a decrease in maintenance requirements for energy [39, 40]. Additionally, in stressful events, herbal supplements minimize stress responses, improving digestibility, absorption, and enzyme secretion [41, 42]. For example, 1, 8-cineole improved FCR and SR in rainbow trout at crowding stress [25]. Similarly, Nile tilapia (*Oreochromis niloticus*) [43] and rainbow trout [44] treated with immunostimulant showed growth parameters elevation even in crowding stress situations. In this study, stocking density did not significantly affect fish survival or mortality.

Under high stocking density, fish can disturb by the production of ROS in excess of body antioxidant capacity [44]. Fish must have a strong antioxidant defense to counteract these negative effects. The high antioxidant compound in herbal extracts can reduce cellular oxidative damage and improve fish immune systems [26, 41]. However, there are no studies based on the ME effect on the antioxidant status of fish under crowding conditions. MDA is a fatty acid peroxidation product and an indicator of oxidative damage in fish [45]. As a result, before the challenge, MDA showed no significant difference among treatments. After the stress, fish fed 1.5% and 2% ME diets presented lower MDA levels than the control group. In line with other investigations, herbal products like lycopene in high-density-stressed rainbow trout caused a dose-dependent decrease in MDA value [44]. Besides, stress caused by high stocking density decrease SOD, GPx, and CAT levels as the first line of an antioxidant defense system. This antioxidant decomposes pro-oxidant molecules [22] as well as catalyzes ROS in less reactive species [46]. Our findings revealed that before the stress, different concentrations of ME significantly elevated SOD, CAT, and GPx. Also, after the crowding stress, dietary ME increased their values. This positive effect may be attributed to the antioxidant effects and phenolic and flavonoid components of medlar as a medicinal herb extract. Also, medlar scavenging H_2_O_2_ in a concentration-dependent manner though indirectly raises the capability to mitigate oxidative stress and resist stress [10]. Liu et al. [47] reported that hepatic SOD, CAT, and GPx levels were depressed in turbot (*Scophthalmus maximus*) held at a high density. Similarly, Xie et al. [48] demonstrated that common carp (*Cyprinus carpio*) fed rhubarb (*Rheum officinale*) had higher hepatic CAT and SOD activities after 1 and 7 days of crowding stress. In another study, 1, 8-cineole (cineole) as an herb essential oil increased serum CAT, SOD, and decreased MDA. Meanwhile, after the stress, MDA decreased and CAT and SOD increased in the treated rainbow trout [25]. The enhancing effects of the plant's extracts on the fish antioxidant system have been reported in many spices like common carp treated with common mallow (*Malvae sylvestris*), oregano (*Origanum vulgare*), and Persian shallot (*Allium hirtifolium* boiss) [49], coriander (*Coriandrum sativum*), common mallow (*M. sylvestris*) fed oak acorn (*Quercus brantii*) [50], bitter melon extract (*Momordica charantia*) [51], Nile tilapia fed thyme powder (*Thymus vulgaris*) [52].

Liver metabolic enzymes are raised in cases of acute injury or dysfunction following disease, stressful situations, and toxicant exposure [53]. ALT and AST are involved in amino acid metabolism and gluconeogenesis [54]. The results of this study demonstrated that in a normal density, high doses of dietary ME modulated ALT and LDH. Also, all ME treatments decreased ALP, and 1.5% of ME showed lower AST levels. In high rearing density, all experimental groups showed lower liver enzyme levels than the control group, which proved the hepatoprotective properties of ME on rainbow trout in stress conditions. Additional studies have indicated that high stocking density decreased hepatosomatic index, altered liver fatty acid composition, and elevated hepatic lipid utilization to increase energy demand [38]. Likewise, Acid and ALP (ACP and AKP) elevation under high stocking density negatively affected fish nonspecific immune response in grass carp (*Ctenopharyngodon idella*) [55]. In addition, the highest and lowest plasma ALP and AST activities were observed in the control and 0.25% herbal material (menthol) treatments, respectively, in rainbow trout [29]. In contrast to the results obtained herein, dietary licorice (*Glycyrrhiza glabra*) supplementations increased ALP activity in 10–30 g/kg licorice groups before and after stress [56]. Further investigations on herbs are needed to prove their action in liver enzymes in stress conditions.

An evaluation of serum immune parameters may demonstrate the potential of herbal additives as promising feed supplements. LYZ is an important natural antimicrobial protein of the innate immune system [57]. It exerts bacteriolytic activity mainly against many Gram-positive bacteria [58, 59]. In our findings, 1% and 1.5% ME immunostimulant increased serum LYZ of rainbow trout. Meanwhile, 1.5% ME could elevate the LYZ level after the challenge. This improvement might be due to an increase in neutrophils, monocytes, and the small number of macrophages [22, 60]. On the other hand, herbal flavonoids stimulate fish leucocytes and PHA generating LYZ secretion [61]. Although high stocking density produced an elevation of LYZ activity compared with the low stocking density group, vitamin C and E dietary ameliorating this effect in gilthead seabream [38]. In addition, common carp fed with 0.5% and 1% anthraquinone extract showed significantly increased in LYZ. After 7 days of stress, the LYZ value in all groups decreased and was restored to the original level [48]. Mirghaed et al. [25] reported that stress significantly decreased serum LYZ activities, whereas *Eucalyptus* sp. EOs supplementation increased its activity in rainbow trout.

Complement proteins, such as C3 and C4, participate in different immune functions, including inflammatory responses, opsonization of pathogens, and clearance of homeostatic cells [62]. The reduction of total complement levels may damage hepatocytes, epithelial cells of the intestine, and mononuclear phagocytes [63]. Our results revealed that the ME supplementation diet didn't affect C3 and C4 levels compared to the control. However, in response to a stress condition, high doses of ME dietary (1.5% and 2%) improved C3 and 1.5% ME modulated C4 value. Based on previous research, complement C3 exhibited no significant difference in the medium and high stocking density of grass carp (*C. idella*), which reflects a reduction tendency in nonspecific immune response [55]. Following these results, an increase in C3 level was reported in Nile tilapia-fed thyme powder [52].

Total Ig is a vital immune constituent and contributes to producing specific antibody responses [64], which are secreted mainly by plasmablasts and plasma cells [65]. In the present study, dietary ME influenced total Ig levels in all treatments. Besides, high doses of supplements increased the level of total Ig in the density-stressed fish. Total Ig elevation may be associated with an increase in B lymphocytes and TP levels to produce opsonizing antibodies [66, 67]. Following these results, all the cineole-treated rainbow trout had significantly higher total Ig compared to the control after the stress [25]. Moreover, dietary plant extracts (Saint John's wort, lemon balm, and rosemary) increase the expression of membrane and secreted immunoglobulin M (IgM) in Atlantic salmon (*Salmo salar*) subjected to crowding stress [67]. Meanwhile, no significant differences in total Ig were found between gilthead seabream held at different densities [38]. Ghafarifarsani et al. [68] reported that the extract of Persian Shallot (*A. hirtifolium*) can regulate the immune system response by affecting the total Ig in Zebrafish (*Danio rerio*).

RBA is an indication of oxidative potential in phagocytes through stimulation by ROS or foreign agents [69]. The present results showed that all ME concentrations increased RBA levels in treated fish. Notably, serum RBA levels were enhanced in fish fed high doses of RBA in crowding density. RBA elevation may be due to the increase in macrophages/monocytes and granulocytes numbers [70]. These findings are in accordance with the previous study's conclusion that RBA activities significantly increased with increasing dietary L-tryptophan supplementations at higher stress densities in sea cucumber (*Apostichopus japonicus*) [71] Also, RBA exhibited a significant increase in Nile tilapia fed *Withania sominefera* root [72].

PRO is an important microbicidal agent that eliminates hydrogen peroxide (H_2_O_2_) [70]. H_2_O_2_ is scavenged by CAT to form water and by PRO to oxygen which participates in the immune defense [73]. Results showed that rainbow trout fed with ME supplements had higher PRO content before and after the challenge. Likewise, PRO levels were increased by dietary supplementation of dietary *β*-glucan (BG) or/and vitamin C in red sea bream (*Pagrus major*) diets [70]. Despite that, European seabass treated with palm fruit extracts alone or in combination with Pdp11 probiotic did not show any significant variations in PRO level throughout the experiment [74]. Also, no statistically significant variations were observed in leucocyte PRO content in gilthead seabream-fed inulin diet [75].

Phagocytes like neutrophils, macrophages, and dendritic cells are recognized to avoid pathogen attacks and eliminate microorganisms in fish immune systems [76]. PHA is usually associated with a respiratory burst which increases the oxidation levels [77]. In the current study, ME administration enhanced PHA activity in T3 before the stress. However, in high density, fish fed T3, T4, and T5 showed higher PHA levels. The present result suggested that plant flavonoids and other bioactive compounds might stimulate leucocytes and PHA, leading to limiting the spread of diseases [61]. Several studies demonstrated that different herbal materials simultaneously increased PHA, such as the stimulatory effects of *Echinacea purpurea* extract [78] and Thai ginseng (*Boesenbergia rotunda*) powder [36] on Nile tilapia; zingerone on white shrimp (*Litopenaeus vannamei*) [79]; and dried lemon peel on Indian major carp (*Labeo rohita*) [80].

GLU is used to estimate the stress response, and it is also the main energy source during starvation [81, 82]. It can be concluded here that 1.5% concentrations of ME in the diet of rainbow trout decrease GLU levels in the pre-challenge period. After the long-term high rearing density, 1.5% and 2% ME diet could decrease GLU compared to the control, which showed stress-relieving and the hypoglycemic effect of ME. High-density stress utilizes glycogen reserves [56]. Meanwhile, in chronic stress, homeostatic mechanisms recover back the GLU to the original level [27]. Likewise, serum GLU levels in fish-fed licorice showed a significant decrease before and after crowding stress compared to the control [56]. Xie et al. [48] reported that after 1 day of stress, herbal medicine could alleviate blood GLU in all test common carp. After 7 days of stress, the GLU in all test groups decreased but was higher than those before stress. Another study demonstrated that after 14 days of stress exposure, the GLU level of stressed Nile tilapia fed the probiotic was higher than that of unstressed fish receiving the probiotic-supplemented feed [43]. In contrast, European seabass stocked at four densities in a submerged net for 1 hr did not show any effect on plasma GLU levels. Also, in that study, there was a significant increase in GLU concentrations in fish held at high stocking density relative to those at low density [37].

Hormonal variations are the first level of the stress reaction [83]. Overcrowding induces a prolonged CORT elevation that stimulates the liver gluconeogenesis and glycogenolysis to release GLU for fish energetic demands [84–86]. In our study, CORT levels obtained in the T4 and T5 were lower than those in the control. However, all treated challenged groups had lower CORT. This trend indicated the modulatory effect of ME constituents to improve fish health during stressful conditions. CORT elevation could be linked either to hypoglycemic hormone (insulin) stimulation or a reduction in GLU absorption [87]. Besides, the hypothalamus–pituitary–interrenal axis will be continuously stimulated to CORT release [88]. Previous studies have shown that stocking density elevates serum CORT in European seabass and gilthead seabream [37, 38]. Common carp, after feeding herbal supplements like thyme essential oil and quercetin [89], showed lower CORT levels. Moreover, dietary *Lippia alba* (*linalool chemotype*) EO in silver catfish (*Rhamdia quelen*) [90], as well as *Myrcia sylvatica* EO in gilthead seabream reduced CORT levels in high stocking density [91].

## 5. Conclusion

The overall data of the current study showed that dietary 2% ME had greater synergistic interactions in stress-related responses of rainbow trout reared in high-density conditions. Therefore, the application of ME could be of interest as feed supplements to enhance the growth and health status and prevent stress-related immunosuppression of rainbow trout under intensive culture. However, further research is recommended to confirm the results obtained.

## Figures and Tables

**Table 1:** Feedstuffs and compositions of the basal diet [22].

Ingredients (g/kg)	Control
Fishmeal^a^	320
Soybean meal (defatted)^b^	260
Wheat flour	173
Meat meal^c^	100
Fish oil	60
Soybean oil	50
Mineral mix^d^	16
Vitamin mix^d^	10
Phytase^e^	3
DL-methionine^f^	3
Proximate composition (%)	
Crude protein	42.50
Crude lipid	16.40
Crude fiber	3.20
Crude ash	9.60

^a^67% protein; 8% lipid. ^b^Gorgan Soya Co., Gorgan, Iran (46% protein). ^c^60% protein; 18% lipid. ^d^The premix provided following amounts per kg of feed: A: 1,000 IU; D3 : 5,000 IU; E: 20 mg; B5 : 100 mg; B2 : 20 mg; B6 : 20 mg; B1 : 20 mg; H: 1 mg; B9 : 6 mg; B12 : 1 mg; B4 : 600 mg; C: 50 mg; Mg: 350 mg; Fe: 13 mg; Co: 2.5 mg; Cu: 3 mg; Zn: 60 mg; Se: 0.3 mg; I: 1.5 mg; Mn: 10 mg). ^e^Golbid Co., Tehran, Iran (10,000 IU). ^f^Mad Tiour Co., Sanandaj, Iran.

**Table 2:** Phenolic content and antioxidant capacities determined by DPPH and *β*-carotene/linoleic acid assays.

ME extract	Test result
Total phenolics mg GAE/g	189.22 ± 3.10
Total flavonoids mg QE/g	57.55 ± 2.15
DPPH % Inhibition percentage	56.77 ± 1.75
*β*-carotene/linoleic acid % oxidative inhibition	74.11 ± 2.40

Results presented as mean ± standard error (*n* = 3).

**Table 3:** Growth parameters of rainbow trout (*Oncorhynchus mykiss*) fed different levels of dietary medlar (*Mespilus germanica* L.) extract) under crowding stress.

Status	Parameters	T1	T2	T3	T4	T5
Before challenge	IW (g)	38.70 ± 0.28^a^	41.27 ± 1.57^a^	40.63 ± 1.32^a^	41.50 ± 1.85^a^	40.30 ± 1.21^a^
	FW (g)	86.40 ± 1.24^b^	89.50 ± 1.44^b^	90.86 ± 1.42^ab^	95.83 ± 1.09^a^	90.16 ± 1.48^ab^
	WG (g)	47.69 ± 1.11^b^	48.22 ± 0.63^ab^	50.23 ± 1.03^ab^	54.33 ± 1.73^a^	49.86 ± 2.00^ab^
	FCR	1.46 ± 0.04^a^	1.39 ± 0.02^ab^	1.32 ± 0.02^ab^	1.24 ± 0.02^b^	1.35 ± 0.04^ab^
	FI (g/day)	1.24 ± 0.01^a^	1.20 ± 0.01^a^	1.18 ± 0.01^a^	1.20 ± 0.01^a^	1.19 ± 0.01^a^
	SGR (%/day)	3.85 ± 0.02^b^	3.91 ± 0.02^ab^	3.94 ± 0.02^ab^	4.03 ± 0.02^a^	3.95 ± 0.03^ab^
	SR (%)	93.00 ± 1.73^a^	96.33 ± 2.02^a^	96.00 ± 0.00^a^	95.33 ± 2.33^a^	95.33 ± 2.90^a^

After challenge	IW (g)	86.40 ± 1.24^b^	89.50 ± 1.44^b^	90.86 ± 1.42^ab^	95.83 ± 1.09^a^	90.16 ± 1.48^ab^
	FW (g)	95.83 ± 1.01^c^	101.00 ± 1.32^bc^	102.66 ± 1.20^b^	108.93 ± 1.18^a^	102.16 ± 1.69^b^
	WG (g)	9.43 ± 0.23^c^	11.50 ± 0.28^b^	11.80 ± 0.30^ab^	13.10 ± 0.43^a^	12.00 ± 0.28^ab^
	FCR	1.72 ± 0.04^a^	1.50 ± 0.02^b^	1.46 ± 0.03^b^	1.38 ± 0.06^b^	1.49 ± 0.04^b^
	FI (g/day)	1.16 ± 0.03^b^	1.23 ± 0.02^ab^	1.23 ± 0.01^ab^	1.29 ± 0.02^a^	1.27 ± 0.01^a^
	SGR (%/day)	0.88 ± 0.03^a^	1.05 ± 0.09^a^	0.95 ± 0.07^a^	1.09 ± 0.02^a^	0.97 ± 0.06^a^
	SR (%)	91.00 ± 1.00^a^	96.33 ± 2.02^a^	95.33 ± 2.90^a^	94.33 ± 2.96^a^	94.33 ± 2.96^a^

Data are expressed as the mean ± SE (*n* = 3). Different letters (a–c) in the same row indicate significant differences among the treatments (*P* < 0.05). T1 (0, control); T2 (1%); T3 (2%); T4 (3%); T5 (4%) ME. IW, initial weight; FW, final weight; WG, weight gain; FCR, feed conversion ratio; FI, feed intake; SGR, specific growth rate; SR, survival rate.

**Table 4:** Serum antioxidant parameters of rainbow trout (*Oncorhynchus mykiss*) fed different levels of dietary medlar (*Mespilus germanica* L.) extract under crowding stress.

Status	Parameters	T1		T2	T3	T4	T5
Before challenge	MDA (*μ*M/l)		8.70 ± 0.63^a^	7.96 ± 0.70^a^	6.03 ± 0.75^a^	5.98 ± 0.73^a^	7.39 ± 0.75^a^
	SOD (U/ml)		37.86 ± 1.91^c^	41.08 ± 2.93^bc^	50.45 ± 1.85^a^	49.65 ± 1.81^ab^	42.75 ± 1.96^abc^
	CAT (U/ml)		15.33 ± 1.18^b^	16.00 ± 1.03^b^	23.91 ± 0.99^a^	23.48 ± 1.23^a^	19.51 ± 1.18^ab^
	GPx (U/ml)		51.16 ± 4.88^c^	67.50 ± 3.78^b^	88.83 ± 4.69^a^	91.83 ± 3.36^a^	77.50 ± 2.04^ab^

After challenge	MDA (*μ*M/l)		12.14 ± 1.08^a^	9.84 ± 1.05^ab^	9.25 ± 0.80^abc^	6.07 ± 0.64^c^	8.07 ± 0.61^bc^
	SOD (U/ml)		34.90 ± 1.15^c^	41.61 ± 2.37^bc^	43.50 ± 2.66^ab^	50.90 ± 1.63^a^	48.43 ± 1.14^ab^
	CAT (U/ml)		14.43 ± 1.04^c^	17.09 ± 1.80^bc^	21.28 ± 1.31^ab^	24.22 ± 1.47^a^	23.32 ± 1.16^a^
	GPx (U/ml)		49.33 ± 3.47^c^	63.16 ± 6.49^bc^	76.33 ± 2.91^ab^	93.00 ± 3.08^a^	89.83 ± 4.30^a^

Data are expressed as the mean ± SE (*n* = 3). Different letters (a–c) in the same row indicate significant differences among the treatments (*P* < 0.05). MDA, malondialdehyde; SOD, superoxide dismutase CAT, catalase; GPx, glutathione peroxidase.

**Table 5:** Serum enzyme of rainbow trout (*Oncorhynchus mykiss*) fed different levels of dietary medlar (*Mespilus germanica* L.) extract under crowding stress.

Status	Parameters	T1	T2	T3	T4	T5
Before challenge	ALT (U/l)	42.72 ± 2.04^a^	37.70 ± 0.95^a^	29.69 ± 1.33^b^	27.71 ± 1.26^b^	31.60 ± 0.83^b^
	AST (U/l)	329.22 ± 9.06^a^	314.66 ± 7.20^ab^	299.83 ± 8.50^ab^	283.83 ± 10.75^b^	293.83 ± 7.85^ab^
	ALP (U/l)	525.50 ± 11.44^a^	436.50 ± 9.03^b^	390.16 ± 6.24^c^	387.16 ± 9.71^c^	433.83 ± 9.03^b^
	LDH (U/l)	956.00 ± 10.16^a^	862.16 ± 10.00^a^	840.16 ± 9.00^b^	795.50 ± 9.75^c^	844.83 ± 8.46^b^

After challenge	ALT (U/l)	51.74 ± 2.36^a^	43.34 ± 1.44^b^	33.60 ± 1.18^c^	28.75 ± 1.20^c^	29.58 ± 1.08^c^
	AST (U/l)	358.27 ± 6.94^a^	318.33 ± 8.08^b^	288.16 ± 9.30^b^	300.16 ± 6.86^b^	301.16 ± 7.44^b^
	ALP (U/l)	579.33 ± 5.98^a^	441.16 ± 4.83^b^	436.00 ± 8.27^b^	390.83 ± 4.87^c^	442.33 ± 8.34^b^
	LDH (U/l)	1036.16 ± 8.58^a^	896.50 ± 8.13^b^	845.83 ± 9.88^c^	796.83 ± 6.24^d^	848.00 ± 6.55^c^

Data are expressed as the mean ± SE (*n* = 3). Different letters (a–e) in the same row indicate significant differences among the treatments (*P* < 0.05). ALT, alanine aminotransferase; AST, aspartate transaminase; ALP, alkaline phosphatase; LDH, lactate dehydrogenase.

**Table 6:** Serum immune parameters of rainbow trout (*Oncorhynchus mykiss*) fed different levels of dietary medlar (*Mespilus germanica* L.) extract under crowding stress.

Status	Parameters	T1	T2	T3	T4	T5
Before challenge	LYZ (U/ml)	20.98 ± 0.89^b^	22.63 ± 0.87^ab^	25.16 ± 1.08^a^	25.30 ± 1.14^a^	23.60 ± 0.95^ab^
	C3 (mg/dl)	19.98 ± 1.02^a^	22.97 ± 0.92^a^	22.40 ± 1.44^a^	24.08 ± 1.33^a^	21.66 ± 1.23^a^
	C4 (mg/dl)	9.02 ± 1.00^a^	9.87 ± 0.97^a^	11.77 ± 0.94^a^	12.74 ± 0.91^a^	9.79 ± 0.84^a^
	Total Ig (mg/ml)	15.75 ± 1.02^b^	20.44 ± 1.12^a^	23.67 ± 1.13^a^	24.31 ± 0.90^a^	21.46 ± 1.14^a^
	RBA (RLU/S)	1,189.83 ± 14.10^c^	1,271.00 ± 21.59^b^	1,423.83 ± 12.59^a^	1,433.33 ± 16.41^a^	1,289.00 ± 13.55^b^
	PRO (*μ*g/ml)	0.09 ± 0.00^c^	0.11 ± 0.00^c^	0.22 ± 0.00^b^	0.27 ± 0.02^a^	0.18 ± 0.01^b^
	PHA (%)	14.50 ± 1.47^b^	15.50 ± 0.76^b^	16.00 ± 3.15^b^	27.66 ± 1.28^a^	18.83 ± 1.32^b^

After challenge	LYZ (U/ml)	17.73 ± 1.38^b^	20.05 ± 1.08^ab^	19.85 ± 1.24^ab^	23.43 ± 1.25^a^	21.50 ± 1.33^ab^
	C3 (mg/dl)	12.40 ± 1.03^b^	12.59 ± 1.14^b^	14.77 ± 1.10^b^	22.87 ± 1.39^a^	20.19 ± 1.19^a^
	C4 (mg/dl)	6.44 ± 0.78^b^	6.62 ± 0.89^b^	7.94 ± 0.71^ab^	11.36 ± 0.99^a^	9.77 ± 0.95^ab^
	Total Ig (mg/ml)	13.06 ± 0.94^d^	15.23 ± 1.01^cd^	18.36 ± 1.08^bc^	23.88 ± 0.89^a^	20.05 ± 1.05^ab^
	RBA (RLU/S)	971.33 ± 22.52^b^	1,043.66 ± 18.76^b^	1,157.16 ± 16.89^a^	1,244.33 ± 24.74^a^	1,190.00 ± 22.50^a^
	PRO (*μ*g/ml)	0.07 ± 0.00^c^	0.09 ± 0.00^c^	0.16 ± 0.01^b^	0.27 ± 0.02^a^	0.19 ± 0.01^b^
	PHA (%)	10.66 ± 0.71^c^	13.50 ± 0.76^bc^	17.00 ± 1.46^b^	27.33 ± 1.28^a^	18.00 ± 1.23^b^

Data are expressed as the mean ± SE (*n* = 3). Different letters (a–e) in the same row indicate significant differences among the treatments (*P* < 0.05). LYZ, lysozyme; C3, the complement protein; C4, the complement protein; Total Ig, total immunoglobulin; RBA, respiratory burst activity; PRO, peroxidase; PHA, phagocytosis.

## Data Availability

The data are available from the corresponding author upon reasonable request.

## References

[1] Umesh M., Santhosh A. S. (2021). A strategic review on use of polyhydroxyalkanoates as an immunostimulant in aquaculture. *Applied Food Biotechnology*.

[2] Ghafarifarsani H., Hoseinifar S. H., Javahery S., Van Doan H. (2022). Effects of dietary vitamin C, thyme essential oil, and quercetin on the immunological and antioxidant status of common carp (*Cyprinus carpio*). *Aquaculture*.

[3] Abdel-Tawwab M., Samir F., El-Naby A. S. A., Monier M. N. (2018). Antioxidative and immunostimulatory effect of dietary cinnamon nanoparticles on the performance of Nile tilapia, *Oreochromis niloticus* (L.) and its susceptibility to hypoxia stress and *Aeromonas hydrophila infection*. *Fish & Shellfish Immunology*.

[4] Reverter M., Bontemps N., Lecchini D., Banaigs B., Sasal P. (2014). Use of plant extracts in fish aquaculture as an alternative to chemotherapy: current status and future perspectives. *Aquaculture*.

[5] Mohan K., Ravichandran S., Muralisankar T. (2019). Potential uses of fungal polysaccharides as immunostimulants in fish and shrimp aquaculture: a review. *Aquaculture*.

[6] Harikrishnan R., Balasundaram C., Heo M.-S. (2011). Impact of plant products on innate and adaptive immune system of cultured finfish and shellfish. *Aquaculture*.

[7] Ferreira F. S., de Oliveira V. S., Chávez D. W. H. (2022). Bioactive compounds of parsley (*Petroselinum crispum*), chives (*Allium schoenoprasum* L) and their mixture (Brazilian *cheiro-verde*) as promising antioxidant and anti-cholesterol oxidation agents in a food system. *Food Research International*.

[8] Bibalani G. H., Mosazadeh-Sayadmahaleh F. (2012). Medicinal benefits and usage of medlar (*Mespilus germanica*) in Gilan province (Roudsar District), Iran. *Journal of Medicinal Plants Research*.

[9] Ayaz F. A., Demir O., Torun H., Kolcuoglu Y., Colak A. (2008). Characterization of polyphenoloxidase (PPO) and total phenolic contents in medlar (*Mespilus germanica* L.) fruit during ripening and over ripening. *Food Chemistry*.

[10] Nabavi S. F., Nabavi S. M., Ebrahimzadeh M. A., Asgarirad H. (2011). The antioxidant activity of wild medlar (*Mespilus germanica* L.) fruit, stem bark and leaf. *African Journal of Biotechnology*.

[11] Gruz J., Ayaz F. A., Torun H., Strnad M. (2011). Phenolic acid content and radical scavenging activity of extracts from medlar (*Mespilus germanica* L.) fruit at different stages of ripening. *Food Chemistry*.

[12] Ghafarifarsani H., Rashidian G., Bagheri T., Hoseinifar S. H., Van Doan H. (2021). Study on growth enhancement and the protective effects of dietary prebiotic inulin on immunity responses of rainbow trout (*Oncorhynchus mykiss*) fry infected with *Aeromonas hydrophila*. *Annals of Animal Science*.

[13] Shahin S., Mishra V., Singh S. P., Chaturvedi C. M. (2014). 2.45-GHz microwave irradiation adversely affects reproductive function in male mouse, *Mus musculus* by inducing oxidative and nitrosative stress. *Free Radical Research*.

[14] Farsani M. N., Hoseinifar S. H., Rashidian G., Farsani H. G., Ashouri G., Van Doan H. (2019). Dietary effects of *Coriandrum sativum* extract on growth performance, physiological and innate immune responses and resistance of rainbow trout (*Oncorhynchus mykiss*) against *Yersinia ruckeri*. *Fish & Shellfish Immunology*.

[15] Adel M., Pourgholam R., Zorriehzahra J., Ghiasi M. (2016). Hemato-immunological and biochemical parameters, skin antibacterial activity, and survival in rainbow trout (*Oncorhynchus mykiss*) following the diet supplemented with *Mentha piperita* against *Yersinia ruckeri*. *Fish & Shellfish Immunology*.

[16] Oroji E., Mehrgan M. S., Islami H. R., Sharifpour I. (2021). Dietary effect of *Ziziphora clinopodioides* extract on zootechnical performance, immune response, and disease resistance against *Yersinia ruckeri* in *Oncorhynchus mykiss*. *Aquaculture Reports*.

[17] da Silva C. P., Soares-Freitas R. A. M., Sampaio G. R. (2019). Identification and action of phenolic compounds of Jatobá-do-cerrado (*Hymenaea stignocarpa* Mart.) on *α*-amylase and *α*-glucosidase activities and flour effect on glycemic response and nutritional quality of breads. *Food Research International*.

[18] Scapin G., Schmidt M. M., Prestes R. C., Rosa C. S. (2016). Phenolics compounds, flavonoids and antioxidant activity of chia seed extracts (*Salvia hispanica*) obtained by different extraction conditions. *International Food Research Journal*.

[19] Fukumoto L. R., Mazza G. (2000). Assessing antioxidant and prooxidant activities of phenolic compounds. *Journal of Agricultural and Food Chemistry*.

[20] Miller H. E. (1971). A simplified method for the evaluation of antioxidants. *Journal of the American Oil Chemists Society*.

[21] Hoseinifar S. H., Khodadadian Zou H., Kolangi Miandare H., Van Doan H., Romano N., Dadar M. (2017). Enrichment of common carp (*Cyprinus carpio*) diet with medlar (*Mespilus germanica*) leaf extract: effects on skin mucosal immunity and growth performance. *Fish & Shellfish Immunology*.

[22] Yousefi M., Shabunin S. V., Vatnikov Y. A. (2020). Effects of lavender (*Lavandula angustifolia*) extract inclusion in diet on growth performance, innate immunity, immune-related gene expression, and stress response of common carp, *Cyprinus carpio*. *Aquaculture*.

[23] AOAC (2005). *Official Methods of Analysis*.

[24] Naderi Farsani M., Meshkini S., Manaffar R. (2021). Growth performance, immune response, antioxidant capacity and disease resistance against *Yersinia ruckeri* in rainbow trout (*Oncorhynchus mykiss*) as influenced through singular or combined consumption of resveratrol and two-strain probiotics. *Aquaculture Nutrition*.

[25] Taheri Mirghaed A., Hoseini S. M., Ghelichpour M. (2018). Effects of dietary 1,8-cineole supplementation on physiological, immunological and antioxidant responses to crowding stress in rainbow trout (*Oncorhynchus mykiss*). *Fish & Shellfish Immunology*.

[26] Hoseinifar S. H., Shakouri M., Yousefi S. (2020). Humoral and skin mucosal immune parameters, intestinal immune related genes expression and antioxidant defense in rainbow trout (*Oncorhynchus mykiss*) fed olive (*Olea europea* L.) waste. *Fish & Shellfish Immunology*.

[27] Naderi M., Keyvanshokooh S., Ghaedi A., Salati A. P. (2019). Interactive effects of dietary Nano selenium and vitamin E on growth, haematology, innate immune responses, antioxidant status and muscle composition of rainbow trout under high rearing density. *Aquaculture Nutrition*.

[28] Góth L. (1991). A simple method for determination of serum catalase activity and revision of reference range. *Clinica Chimica Acta*.

[29] Hoseini S. M., Hoseinifar S. H., Van Doan H. (2021). Growth performance and hematological and antioxidant characteristics of rainbow trout, *Oncorhynchus mykiss*, fed diets supplemented with Roselle, *Hibiscus sabdariffa*. *Aquaculture*.

[30] Adineh H., Harsij M., Jafaryan H., Asadi M. (2020). The effects of microencapsulated garlic (*Allium sativum*) extract on growth performance, body composition, immune response and antioxidant status of rainbow trout (*Oncorhynchus mykiss*) juveniles. *Journal of Applied Animal Research*.

[31] Ellis A. E., Stolen J. S., Fletcher T. C., Anderson D. P., Robertson B. S. V., Muiswinkel W. R. (1990). *Lysozyme Assay 299 in Techniques in Fish Immunology*.

[32] Binaii M., Ghiasi M., Farabi S. Mohammad V. (2014). Biochemical and hemato-immunological parameters in juvenile beluga (*Huso huso*) following the diet supplemented with nettle (*Urtica dioica*). *Fish & Shellfish Immunology*.

[33] Cordero H., Cuesta A., Meseguer J., Ángeles Esteban M. (2016). Changes in the levels of humoral immune activities after storage of gilthead seabream (*Sparus aurata*) skin mucus. *Fish & Shellfish Immunology*.

[34] Zhou X., Niu C., Sun R., Li Q. (2022). The effect of vitamin C on the non-specific immune response of the juvenile soft-shelled turtle (Trionyx sinensis). *Comparative Biochemistry and Physiology Part A: Molecular & Integrative Physiology*.

[35] Ortuño J., Esteban M. A., Meseguer J. (2001). Effects of short-term crowding stress on the gilthead seabream (*Sparus aurata* L.) innate immune response. *Fish & Shellfish Immunology*.

[36] Van Doan H., Hoseinifar S. H., Chitmanat C. (2019). The effects of Thai ginseng, *Boesenbergia rotunda* powder on mucosal and serum immunity, disease resistance, and growth performance of Nile tilapia (*Oreochromis niloticus*) fingerlings. *Aquaculture*.

[37] Santos G. A., Schrama J. W., Mamauag R. E. P., Rombout J. H. W. M., Verreth J. A. J. (2010). Chronic stress impairs performance, energy metabolism and welfare indicators in European seabass (*Dicentrarchus labrax*): the combined effects of fish crowding and water quality deterioration. *Aquaculture*.

[38] Montero D., Izquierdo M. S., Tort L., Robaina L., Vergara J. M. (1999). High stocking density produces crowding stress altering some physiological and biochemical parameters in gilthead seabream, *Sparus aurata*, juveniles. *Fish Physiology and Biochemistry*.

[39] Aguilar V., Racotta I. S., Goytortúa E. (2012). The influence of dietary arachidonic acid on the immune response and performance of pacific whiteleg shrimp, *Litopenaeus vannamei*, at high stocking density. *Aquaculture Nutrition*.

[40] Mzengereza K., Ishikawa M., Koshio S. (2021). Growth performance, growth-related genes, digestibility, digestive enzyme activity, immune and stress responses of *de novo* camelina meal in diets of red seabream (*Pagrus major*). *Animals*.

[41] Souza C. D. F., Baldissera M. D., Baldisserotto B., Heinzmann B. M., Martos-Sitcha J. A., Mancera J. M. (2019). Essential oils as stress-reducing agents for fish aquaculture: a review. *Frontiers in Physiology*.

[42] Xu D., Hu M.-J., Wang Y.-Q., Cui Y.-L. (2019). Antioxidant activities of quercetin and its complexes for medicinal application. *Molecules*.

[43] Gonçalves A. T., Maita M., Futami K., Endo M., Katagiri T. (2011). Effects of a probiotic bacterial *Lactobacillus rhamnosus* dietary supplement on the crowding stress response of juvenile Nile tilapia *Oreochromis niloticus*. *Fisheries Science*.

[44] Sahin K., Yazlak H., Orhan C., Tuzcu M., Akdemir F., Sahin N. (2014). The effect of lycopene on antioxidant status in rainbow trout (*Oncorhynchus mykiss*) reared under high stocking density. *Aquaculture*.

[45] Parvez S., Raisuddin S. (2005). Protein carbonyls: novel biomarkers of exposure to oxidative stress-inducing pesticides in freshwater fish *Channa punctata* (Bloch). *Environmental Toxicology and Pharmacology*.

[46] Tang Q. Q., Feng L., Jiang W. D. (2013). Effects of dietary copper on growth, digestive, and brush border enzyme activities and antioxidant defense of hepatopancreas and intestine for young grass carp (*Ctenopharyngodon idella*). *Biological Trace Element Research*.

[47] Liu B., Jia R., Han C., Huang B., Lei J.-L. (2016). Effects of stocking density on antioxidant status, metabolism and immune response in juvenile turbot (*Scophthalmus maximus*). *Comparative Biochemistry and Physiology Part C: Toxicology & Pharmacology*.

[48] Xie J., Liu B., Zhou Q. (2008). Effects of anthraquinone extract from rhubarb *Rheum officinale* Bail on the crowding stress response and growth of common carp *Cyprinus carpio* var. *Jian*. *Aquaculture*.

[49] Ghafarifarsani H., Hoseinifar S. H., Adorian T. J., Ferrigolo F. R. G., Raissy M., Van Doan H. (2021). The effects of combined inclusion of *Malvae sylvestris*, *Origanum vulgare*, and *Allium hirtifolium* boiss for common carp (*Cyprinus carpio*) diet: growth performance, antioxidant defense, and immunological parameters. *Fish & Shellfish Immunology*.

[50] Raissy M., Ghafarifarsani H., Hoseinifar S. H., El-Haroun E. R., Shahbazi Naserabad S., Van Doan H. (2022). The effect of dietary combined herbs extracts (oak acorn, coriander, and common mallow) on growth, digestive enzymes, antioxidant and immune response, and resistance against *Aeromonas hydrophila* infection in common carp, *Cyprinus carpio*. *Aquaculture*.

[51] Qin C., Wang J., Zhao W., Pi D., Yan X., Nie G. (2022). Effects of dietary bitter melon extract on growth performance, antioxidant capacity, inflammatory cytokines expression, and intestinal microbiota in common carp (*Cyprinus carpio* L.). *Aquaculture Nutrition*.

[52] Khalil S. R., Elhakim Y. A., Abd El-fattah A. H., Ragab Farag M., Abd El-Hameed N. E., EL-Murr A. E. (2020). Dual immunological and oxidative responses in *Oreochromis niloticus* fish exposed to lambda cyhalothrin and concurrently fed with Thyme powder (*Thymus vulgaris* L.): stress and immune encoding gene expression. *Fish & Shellfish Immunology*.

[53] Usunomena U., Ademuyiwa A. J., Okugbo Tinuade O., Evuen Uduenevwo F., Martin O., Okolie N. P. (2012). N-nitrosodimethylamine (NDMA), liver function enzymes, renal function parameters and oxidative stress parameters: a review. *British Journal of Pharmacology and Toxicology*.

[54] Molina R., Moreno I., Pichardo S. (2005). Acid and alkaline phosphatase activities and pathological changes induced in Tilapia fish (*Oreochromis* sp.) exposed subchronically to microcystins from toxic cyanobacterial blooms under laboratory conditions. *Toxicon*.

[55] Lin W., Li L., Chen J. (2018). Long-term crowding stress causes compromised nonspecific immunity and increases apoptosis of spleen in grass carp (*Ctenopharyngodon idella*). *Fish & Shellfish Immunology*.

[56] Adineh H., Naderi M., Yousefi M., Khademi Hamidi M., Ahmadifar E., Hoseini S. M. (2021). Dietary licorice (*Glycyrrhiza glabra*) improves growth, lipid metabolism, antioxidant and immune responses, and resistance to crowding stress in common carp, *Cyprinus carpio*. *Aquaculture Nutrition*.

[57] Zhang C.-N., Li X.-F., Xu W.-N. (2013). Combined effects of dietary fructooligosaccharide and *Bacillus licheniformis* on innate immunity, antioxidant capability and disease resistance of triangular bream (*Megalobrama terminalis*). *Fish & Shellfish Immunology*.

[58] Ibrahim H. R., Yamada M., Matsushita K., Kobayashi K., Kato A. (1994). Enhanced bactericidal action of lysozyme to escherichia coli by inserting a hydrophobic pentapeptide into its C terminus. *Journal of Biological Chemistry*.

[59] Abdel-Latif M. A., El-Far A. H., Elbestawy A. R., Ghanem R., Mousa S. A., Abd El-Hamid H. S. Exogenous dietary lysozyme improves the growth performance and gut microbiota in broiler chickens targeting the antioxidant and non-specific immunity mRNA expression. *PLoS ONE*.

[60] Nhu T. Q., Bich Hang B. T., Vinikas A. (2019). Screening of immuno-modulatory potential of different herbal plant extracts using striped catfish (*Pangasianodon hypophthalmus*) leukocyte-based *in vitro* tests. *Fish & Shellfish Immunology*.

[61] Awad E., Cerezuela R., Esteban M.Á. (2015). Effects of fenugreek (*Trigonella foenum graecum*) on gilthead seabream (*Sparus aurata* L.) immune status and growth performance. *Fish & Shellfish Immunology*.

[62] Holland M. C. H., Lambris J. D. (2002). The complement system in teleosts. *Fish & Shellfish Immunology*.

[63] Banaee M., Soltanian S., Sureda A. (2019). Evaluation of single and combined effects of cadmium and micro-plastic particles on biochemical and immunological parameters of common carp (*Cyprinus carpio*). *Chemosphere*.

[64] Tarkhani R., Imani A., Hoseinifar S. H. (2020). Comparative study of host-associated and commercial probiotic effects on serum and mucosal immune parameters, intestinal microbiota, digestive enzymes activity and growth performance of roach (*Rutilus rutilus caspicus*) fingerlings. *Fish & Shellfish Immunology*.

[65] Esteban A. M. (2012). *An Overview of the Immunological Defenses in Fish Skin*.

[66] Ercal N., Neal R., Treeratphan P. (2000). A role for oxidative stress in suppressing serum immunoglobulin levels in lead-exposed fisher 344 rats. *Archives of Environmental Contamination and Toxicology*.

[67] Reyes-Cerpa S., Vallejos-Vidal E., Gonzalez-Bown M. J. (2018). Effect of yeast (*Xanthophyllomyces dendrorhous*) and plant (Saint John’s wort, lemon balm, and rosemary) extract based functional diets on antioxidant and immune status of Atlantic salmon (*Salmo salar*) subjected to crowding stress. *Fish & Shellfish Immunology*.

[68] Ghafarifarsani H., Hoseinifar S. H., Talebi M. (2021). Combined and singular effects of ethanolic extract of Persian shallot (*Allium hirtifolium Boiss*) and synbiotic Biomin® IMBO on growth performance, serum-and mucus-immune parameters and antioxidant defense in Zebrafish (*Danio rerio*). *Animals*.

[69] Akhter N., Wu B., Memon A. M., Mohsin M. (2015). Probiotics and prebiotics associated with aquaculture: a review. *Fish & Shellfish Immunology*.

[70] Dawood M. A. O., Koshio S., El-Sabagh M. (2017). Changes in the growth, humoral and mucosal immune responses following *β*-glucan and vitamin C administration in red sea bream, *Pagrus major*. *Aquaculture*.

[71] Zhang E., Dong S., Wang F., Tian X., Gao Q. (2018). Effects of L-tryptophan on the growth, intestinal enzyme activities and non-specific immune response of sea cucumber (*Apostichopus japonicus* Selenka) exposed to crowding stress. *Fish & Shellfish Immunology*.

[72] Zahran E., Abd El-Gawad E. A., Risha E. (2018). Dietary *Withania sominefera* root confers protective and immunotherapeutic effects against *Aeromonas hydrophila* infection in Nile tilapia (*Oreochromis niloticus*). *Fish & Shellfish Immunology*.

[73] Holmblad T., Söderhäll K. (1999). Cell adhesion molecules and antioxidative enzymes in a crustacean, possible role in immunity. *Aquaculture*.

[74] Guardiola F. A., Porcino C., Cerezuela R., Cuesta A., Faggio C., Esteban M. A. (2016). Impact of date palm fruits extracts and probiotic enriched diet on antioxidant status, innate immune response and immune-related gene expression of European seabass (*Dicentrarchus labrax*). *Fish & Shellfish Immunology*.

[75] Cerezuela R., Cuesta A., Meseguer J., Ángeles Esteban M. (2008). Effects of inulin on gilthead seabream (*Sparus aurata* L.) innate immune parameters. *Fish & Shellfish Immunology*.

[76] Carbone D., Faggio C. (2016). Importance of prebiotics in aquaculture as immunostimulants. Effects on immune system of *Sparus aurata* and *Dicentrarchus labrax*. *Fish & Shellfish Immunology*.

[77] Song Y.-L., Hsieh Y.-T. (1994). Immunostimulation of tiger shrimp (*Penaeus monodon*) hemocytes for generation of microbicidal substances: analysis of reactive oxygen species. *Developmental & Comparative Immunology*.

[78] Abdel Rahman A. N., Khalil A. A., Abdallah H. M., ElHady M. (2018). The effects of the dietary supplementation of *Echinacea purpurea* extract and/or vitamin C on the intestinal histomorphology, phagocytic activity, and gene expression of the Nile tilapia. *Fish & Shellfish Immunology*.

[79] Chang Y.-P., Liu C.-H., Wu C.-C., Chiang C.-M., Lian J.-L., Hsieh S.-L. (2012). Dietary administration of zingerone to enhance growth, non-specific immune response, and resistance to *Vibrio alginolyticus* in pacific white shrimp (*Litopenaeus vannamei*) juveniles. *Fish & Shellfish Immunology*.

[80] Harikrishnan R., Thamizharasan S., Devi G. (2020). Dried lemon peel enriched diet improves antioxidant activity, immune response and modulates immuno-antioxidant genes in *Labeo rohita* against *Aeromonas sorbia*. *Fish & Shellfish Immunology*.

[81] Kwan B. K. Y., Chan A. K. Y., Cheung S. G., Shin P. K. S. (2014). Hemolymph quality as indicator of health status in juvenile Chinese horseshoe crab *Tachypleus tridentatus* (Xiphosura) under laboratory culture. *Journal of Experimental Marine Biology and Ecology*.

[82] Zhang Y., Xiao X., Feng H., Nikitina M. A., Zhang X., Zhao Q. (2023). Stress fusion evaluation modeling and verification based on non-invasive blood glucose biosensors for live fish waterless transportation. *Frontiers in Sustainable Food Systems*.

[83] Wendelaar Bonga S. E. (1997). The stress response in fish. *Physiological Reviews*.

[84] Sheridan M. A. (1986). Effects of thyroxin, cortisol, growth hormone, and prolactin on lipid metabolism of coho salmon, *Oncorhynchus kisutch*, during smoltification. *General and Comparative Endocrinology*.

[85] Pickering A. D., Pottinger T. G. (1989). Stress responses and disease resistance in salmonid fish: effects of chronic elevation of plasma cortisol. *Fish Physiology and Biochemistry*.

[86] Hontela A., Rasmussen J. B., Chevalier G. (1993). Endocrine responses as indicators of sublethal toxic stress in fish from polluted environments. *Water Quality Research Journal*.

[87] El Basuini M. F., Shahin S. A., Teiba I. I. (2021). The influence of dietary coenzyme Q10 and vitamin C on the growth rate, immunity, oxidative-related genes, and the resistance against Streptococcus agalactiae of Nile tilapia (*Oreochromis niloticus*). *Aquaculture*.

[88] McCormick S. D., Shrimptom J. M., Carey J. B. (1998). Repeated acute stress reduces growth rate of Atlantic salmon parr and alters plasma levels of growth hormone, insulin-like growth factor I and cortisol. *Aquaculture*.

[89] Ghafarifarsani H., Hoseinifar S. H., Javahery S., Yazici M., Van Doan H. (2022). Growth performance, biochemical parameters, and digestive enzymes in common carp (*Cyprinus carpio*) fed experimental diets supplemented with vitamin C, thyme essential oil, and quercetin. *Italian Journal of Animal Science*.

[90] Souza C. D. F., Salbego J., Gressler L. T. (2015). *Rhamdia quelen* (Quoy & Gaimard, 1824), submitted to a stressful condition: effect of dietary addition of the essential oil of *Lippia alba* on metabolism, osmoregulation and endocrinology. *Neotropical Ichthyology*.

[91] Saccol E. M. H., Parrado-Sanabria Y. A., Gagliardi L. (2018). *Myrcia sylvatica* essential oil in the diet of gilthead sea bream (*Sparus aurata* L.) attenuates the stress response induced by high stocking density. *Aquaculture Nutrition*.

[92] Yousefi M., Ghafarifarsani H., Hoseini S. M. (2022). Effects of dietary thyme essential oil and prebiotic administration on rainbow trout (*Oncorhynchus mykiss*) welfare and performance. *Fish & Shellfish Immunology*.

